# The Passo Fundo Cohort Study: design of a population-based observational study of women in premenopause, menopausal transition, and postmenopause

**DOI:** 10.1186/s40695-015-0013-8

**Published:** 2015-12-18

**Authors:** Karen Oppermann, Verônica Colpani, Sandra C. Fuchs, Poli Mara Spritzer

**Affiliations:** 1 School of Medicine, Passo Fundo University, Passo Fundo, RS Brazil; 2 Hospital São Vicente de Paulo, Passo Fundo, RS Brazil; 3 Gynecological Endocrinology Unit, Division of Endocrinology, Hospital de Clinicas de Porto Alegre, Porto Alegre, RS Brazil; 4 Department of Social Medicine, School of Medicine, Federal University of Rio Grande do Sul, Porto Alegre, RS Brazil; 5 Department of Physiology, Federal University of Rio Grande do Sul, Porto Alegre, RS Brazil

**Keywords:** Menopause, Cohort studies, Central adiposity, Cardiovascular risk factors, Cardiovascular events, Ovarian volume, Coronary artery calcium, Bone mineral density

## Abstract

**Background:**

The Passo Fundo Cohort Study (PFS) is a population-based longitudinal observational study of pre-, peri-, and postmenopausal women that has been ongoing since 1995 in Passo Fundo, a city in southern Brazil. This paper describes the rationale and design of the PFS and summarizes objectives and procedures that have been updated during follow-up.

**Methods/Design:**

Women in the PFS have been followed for a variety of diseases that are frequent in menopause. Sampling was conducted in 154 randomly selected census divisions (geographical subdivisions of the city as defined by the Brazilian Institute of Geography and Statistics). One block in each census division was chosen by lot and two women were randomly selected for interview in each block. The first cycle, conducted between 1995 and 1997, included a representative sample of 298 women aged 35 to 55 years. In the second cycle, conducted between 2001 and 2002, additional participants were enrolled based on the same sampling strategy used in 1995, for a final sample of 358 women. In 2010, a third follow-up was initiated, when all 358 participants or their relatives were located. Participants completed a standardized questionnaire on demographic and socioeconomic characteristics. They also answered questions about lifestyle, medical and reproductive characteristics, sexual life, hormone therapy and mental aspects by using validated instruments. Physical activity was assessed and anthropometric measurements, blood sampling and pelvic ultrasound examination were performed. In the third cycle, bone mineral density by dual-energy X-ray absorptiometry and abdominal fat and coronary artery calcium score by computed tomography were also determined.

**Discussion:**

The study findings provide relevant information to evaluate the association between menopausal status, female aging and the risk of cardiovascular diseases, and bone health aspects in a representative sample of women from southern Brazil.

## Background

Improvements in health care and sanitation have reduced mortality from infectious diseases in developing countries. As a consequence, non-communicable diseases, such as diabetes and cardiovascular disease, are now the leading cause of morbidity and mortality worldwide, with a huge impact on health and society [1, 2]. In Brazil, an upper-middle-income country, only a few observational studies have examined non-communicable diseases, especially regarding women’s health [3]. In this respect, the Passo Fundo Cohort Study (PFS) has been conducted for nearly 15 years, investigating pre-, peri-, and postmenopausal women. This paper describes the rationale and design of the PFS and summarizes objectives and procedures that have been updated during follow-up.

The main objective of the first cycle of the PFS was to investigate the prevalence of climacteric symptoms and their association with transvaginal sonographic features and hormone levels in pre- and perimenopausal women. It also investigated lifestyle habits, socioeconomic status, sexual activity, menstrual complaints, and hormone therapy in this population.

In the second cycle, the main goal of the study was to investigate the association of obesity, central adiposity, and ovarian volume with menopausal status, taking into account several confounding factors. In addition, the relationship between physical, psychological, and menopause-related symptoms and minor psychiatric disorders was also studied.

In the third cycle, the objective was to assess cardiovascular risk among pre-, peri-, and postmenopausal women through habitual physical activity, coronary artery calcium, abdominal fat and anthropometric measurements.

Regarding the cohort design, the aim was to assess clinical, hormonal, and metabolic features and bone mass in these women over time in relation to cardiovascular risk and prediction of cardiovascular events.

## Methods/design

### The design of the Passo Fundo Cohort Study (PFS)

**Fig. 1:**
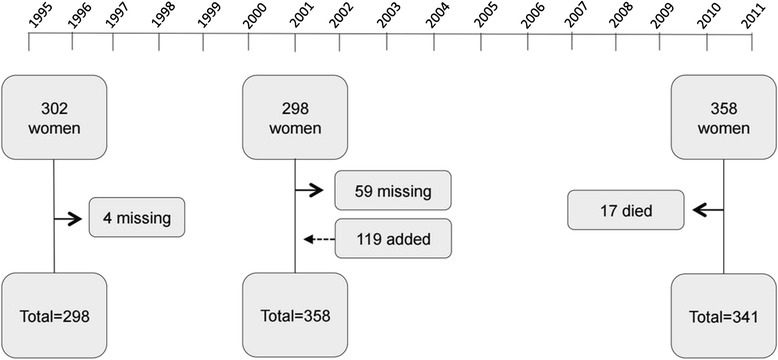
Cycles of the Passo Fundo Cohort Study (PFS). Year 1995–1996 (first cycle): baseline assessment of the original cohort. Year 2001–2002 (second cycle): baseline reassessment and cohort expansion to include 119 additional women aged 35-62 years. Year 2010–2011 (third cycle): first reassessment of women from the second cycle. Continuous arrows indicate missing or dead/deceased women. Dotted arrow indicates added women. Modified from Colpani et al. 2014 [33]

The PFS is a prospective cohort study conducted in Passo Fundo, a city in southern Brazil. The sample consists of women in pre-, peri-, and postmenopause. Figure 1 shows a flow chart of the three cycles of the study.

#### First cycle

From January 1996 to February 1997, participants were recruited for the study. The sample for the first study was selected from the 16,958 women between 35 and 55 years of age living in Passo Fundo according to the 1991 census. Women with an intact uterus and at least one period in the previous 12 months were invited to participate. Sampling was conducted in two stages. First, 154 geographical subdivisions of the city, defined by the Brazilian Institute of Geography and Statistics [4] as census divisions, were randomly selected. One block in each census division was chosen by lot and two women were randomly selected for interview in each block according to the following procedures: in all blocks, each corner was classified as A, B, C, or D, following the same order from left to right and from bottom to top. To start the interviews, one block was chosen per census division, and one corner per block. Two women were then interviewed in each block. When the drawn corner was A or B, the first woman interviewed was chosen for blood sampling and ultrasound examination; when the drawn corner was C or D, the second woman interviewed was chosen for examination. From the selected corner, the block was investigated in a clockwise direction until an eligible participant was found. Once found, the two nearby houses were excluded, and the third one was screened. If there was more than one eligible woman per household, the one with the most recent birthday was selected for the interview. If the potential participant was not at home, the interviewers returned to the household on another day to interview her. If the selected household was a building, only one interview was performed; the floor and apartment for the interview were also randomly selected. A loss was considered to have occurred when women refused to participate in the study or to undergo ultrasound examination after three attempts by the interviewer. A sample of 302 women aged 35 to 55 years who had had at least one period in the previous 12 months was randomly selected. At the end of the first cycle (1995–1996), a representative sample of 298 women was interviewed (response rate 83.2 %).

#### Second cycle

A second home visit was conducted between July 2001 and July 2002, when 239 women of the baseline cohort were located and interviewed [5, 6]. The investigators searched for information about first cycle participants with neighbors, in electricity and water company databases, in medical records at Hospital São Vicente de Paulo, and at the Passo Fundo University, the municipal health department, and the Health Information Center (NIS/RS-SES). The retention percent was 80.2 %. Losses were classified as follows: unwilling to participate (19 = 6.37 %), not found (21 = 7 %), moved out of the city (15 = 5 %), and died (4 = 1.3 %).

In view of potential losses to follow-up and considering an increase in population size, 120 additional women aged 35 to 62 years were randomly selected to ensure sufficient statistical power for analysis. Of these, 119 were interviewed (participation percent 99 %, final total sample of 358 participants). The new participants were randomly selected based on the 175 census divisions defined in the 2000 IBGE census. Sixty new census divisions were chosen by lot and one block was chosen per division, following the same randomization procedures used in the first cycle. Eligibility did not include menstrual criteria for this additional sample of women, since some women from the first cycle had already reached postmenopausal status.

#### Third cycle

In 2010, a third follow-up was initiated in order to assess cardiovascular outcomes [7]. Every effort was made to locate each participant of the previous cycle. For that, contact was made with close relatives, neighbors, and neighboring households; in addition, we reviewed inpatient and outpatient records at Hospital São Vicente and University of Passo Fundo, municipal department of health records in search of women registered to receive medication through the public health system SUS, the city dental registry and the city death records. We also ran an advertisement campaign on two radio stations, Planalto and Uirapuru, reaching 60 municipalities and around 30 thousand listeners per day, and two television stations, RBS TV Passo Fundo and UPF TV, reaching 85 municipalities. All 358 participants or their relatives were located and information on the participant’s vital status was obtained for the period ending in November 2011.

#### Setting

Passo Fundo is a city located in Rio Grande do Sul, the southernmost state of Brazil. The city has a current population of approximately 184 000 inhabitants (data from the 2010 IBGE census) [8]. The economy is based primarily on agriculture and business [8].

### Population

The representative sample included in the PFS consists of pre-, peri-, and postmenopausal women living in the urban area of Passo Fundo, although, in the beginning of the study, the sample was composed only of pre- and perimenopausal women. Menopausal status was determined based on the characteristics of menses or time since amenorrhea: premenopause was defined as no change in menstrual frequency or flow, and perimenopause was defined as changes in menstrual frequency or flow in the 12 months before the study; and postmenopause was defined as 12 or more months of amenorrhea occurring naturally or because of surgical intervention, such as bilateral oophorectomy. Questions such as “when was your last period?”, “how is your menstrual flow?” and “ have you ever been submitted to gynecological surgery (in which organ)?” were asked. A “hysterectomy” category was created for women who had previously undergone hysterectomy without bilateral oophorectomy and whose menopausal status could not be classified.

#### Eligibility criteria

The PFS was designed as a longitudinal study in order to analyze pre-, peri-, and postmenopausal women. Initially, eligible participants were women aged 35 to 55 years who had at least one menstrual cycle in the past 12 months and who were living in the urban area of Passo Fundo, Brazil.

#### Exclusion criteria

Women who had undergone bilateral oophorectomy and/or hysterectomy were excluded from the study. An eligible participant was considered a loss if she refused to participate in the study after three attempts of having her accept participation.

### Sample size

In the first cycle, the sample size was based on a population of 16,958 women between 35 and 55 years of age. The sample size for prevalence studies was calculated based on the assumption that 20 % of premenopausal and perimenopausal women would have climacteric symptoms, with a 15 % error and 95 % CI. The sample size was increased by an additional 10 % to account for potential missing data, for a targeted sample size of 318 women.

For the second cycle, the sample size was estimated based on population growth for year 2000. The estimated population growth was 2.03 % per year between 1991-1996, and 1.88 % per year between 1996 and 2000. Therefore, the growth in this period was 17.7 %. Based on this information, 54 women were randomly selected. Sixty-six additional women were selected to cover potential follow-up losses. The power of the study was thus maintained.

### Ethical considerations

The PFS was approved by the institutional review board of both Passo Fundo University and Hospital de Clínicas de Porto Alegre. Written informed consent was obtained from each participant. During data collection, care was taken to ensure confidentiality of participants’ information.

### Methods

A standardized questionnaire covering demographic characteristics (age and self-reported skin color), education, medical history, family history, reproductive history, sexual activity (in the last three months), lifestyle/behavioral factors, quality of life, medication/supplement inventory, climacteric symptoms, and hormone therapy was applied. More details regarding which items were included in each cycle are shown in Table 1. In the first and second cycles, participants were interviewed at home and examinations were performed at Hospital São Vicente de Paulo. In the third cycle, all measurements and interviews were conducted at the hospital.

**Table 1:** Questionnaires and clinical information assessed in the PFS

Variable	Method	Cycle (year)
Clinical and demographic characteristics	Self-report	1995/2001/2010
Physical activity		
	Self-report	1995
	Modifiable Activity Questionnaire (MAQ) [19]	2001
	International Physical Activity Questionnaire (IPAQ) [22]	2010
	Pedometer	2010
Quality of life		
	Women’s Health Questionnaire (WHQ) [12]	2010
	12-Item Health Survey (SF-12) [14]	2010
Menopausal symptoms		
	Kupperman Menopausal Index [11]	1995/2001/2010
Psychiatric disorders		
	20-Item Self-Reporting Questionnaire (SRQ-20) [17]	2001/2010
Blood samples		
	Hormones	1995/2001/2010
	Cholesterol (mg/dL) (mmol/L)	2001/2010
	Triglycerides (mg/dL) (mmol/L)	2001/2010
	Glucose (mg/dL) (mmol/L)	2001/2010
	Insulin (mU/L)	2001/2010
	DNA samples	2010
Anthropometric measurements		
	Body mass index	1995/2001/2010
	Waist circumference	2001/2010
	Waist-to-hip ratio	2001/2010
Skinfolds	Calipers	2001
Diagnostic imaging		
	Pelvic Ultrasound	1995/2001
	Densitometry	2010
	Computed tomography	2010

#### Training

Field training of students and health care professionals was conducted by the PFS coordinators according to the study protocol (interviewer guide). Intra and inter observer reliability was determined. All participants were thoroughly examined at baseline.

A pilot study was conducted in July 1995. The main objective of this pilot study was to test the agreement between ultrasound operators; additional objectives were to probe the participants’ understanding of questions and to monitor adherence to hospital visits for collection of blood samples and ultrasound examination. Fifty-six premenopausal women were randomly enrolled from 14 census divisions. Two women were excluded due to total hysterectomy and bilateral oophorectomy. During the pilot study, pelvic ultrasound was performed by two examiners, resulting in 83 % agreement between them. Interviews were tested for reliability and reproducibility. Interobserver reliability for anthropometric and blood pressure measurements was also verified by repeated measurements during the first set of consultations (R values were higher than 0.90, *p* < 0.05).

### Study variables

#### Education

Educational attainment was assessed through the number of years of successful formal education, described as years at school.

#### Socioeconomic status

Socioeconomic status was assessed using an instrument developed by the Brazilian Association of Market Research Institutes (Associação Brasileira de Institutos de Pesquisa de Mercado, ABIPEME) [9]. It classifies individuals into five socioeconomic groups: A, B, C, D, and E, where A represents the highest socioeconomic level. This classification is based on 10 selected variables: level of education of the household head, number of cars, having a washing machine and videotape recorder, having a vacuum cleaner, having a refrigerator, number of color televisions, number of bathrooms, number of radios, and being able to pay a housekeeper. The participants were classified as working or not working, and by employment status (employer, employee, self-employed, housemaker).

#### Alcohol intake

From the second cycle onward, alcohol consumption was determined by self-reported alcohol intake (non-drinker or former drinker). In 2001 and 2010, participants were asked what type of alcohol (wine, beer, spirits) they consumed and frequency of consumption.

#### Smoking

Smoking status was also assessed from the second cycle onward. Participants were considered smokers if they smoked more than five cigarettes per day [10]. Subsequently, in 2010, the amount of smoking and age at smoking onset and cessation were assessed in detail using a self-administered questionnaire. Former smokers were defined as individuals who reported having quit smoking and, in this case, they were also asked how long it had been since they quit smoking. No minimum time since smoking cessation was employed in the definition of former smokers. Because there was no specific question on occasional smoking, occasional smokers were grouped as current or former smokers if they met the aforementioned criteria.

#### Gynecological data

Women were asked questions about age at menarche, delivery history (vaginal delivery, cesarean delivery, miscarriage), previous gynecological surgery, and sexual activity (in the last three months). Use of oral contraceptive, hormone therapy, estrogen, estrogen plus progestin or tibolone was verified by asking the participants to show the medication box or the physician’s prescription and categorized according to duration (in years) of use.

#### Menopausal symptoms

The Kupperman Menopausal Index was used to evaluate menopausal symptoms [11]. It is a numerical index that ranks menopausal symptoms based on the sum of scores attributed to variables according to the presence and intensity of symptoms. Scores range from 0 to 3, where 0 = absent, 1 = mild, 2 = moderate, and 3 = intense symptoms. Weighted values were assigned to the variables, as follows: hot flashes (4), night sweats (4), vaginal dryness (3), insomnia (2), memory (2), nervousness (2), depression (1), dizziness (1), fatigue (1), and headache (1).

### Clinical history

#### Blood draw

At baseline, blood was collected from a subsample of 140 women. Blood samples were collected on the same day of the ultrasound examination, between 4 and 6 PM. The day of the reproductive cycle was recorded on this occasion; therefore, tests were performed on any day of the menstrual cycle. In the second and third study cycles, blood samples were collected from all participants between 8 and 10 AM after an overnight fast of 10 to 12 h. In the third study cycle (2010), blood was also drawn and stored at −80 °C for future hormone studies, and DNA was extracted from blood samples and stored for future genetic and molecular analysis.

#### Blood pressure measurements

Standardized blood pressure measurements were performed twice, using the same calibrated mercury manometer. The average of two assessments was used in the analysis. Hypertension was identified by systolic blood pressure greater than or equal to 140 mm Hg or diastolic blood pressure greater than or equal to 90 mm Hg.

#### Hypertension

At baseline, hypertension was assessed by asking about previous diagnosis of hypertension and measuring blood pressure during the home interview. In the second and third cycles, the question about previous diagnosis was asked again and blood pressure was measured at the clinic. Family history of hypertension (parents and grandparents) was investigated from the second cycle (2001) onward.

#### Dyslipidemia

In the three cycles, self-reported hypercholesterolemia and use of anti-cholesterol drugs were used to define dyslipidemia. Blood tests were also performed to measure cholesterol and triglycerides levels.

#### Diabetes

Diabetes was determined by self-report, use of antidiabetic drugs, or a fasting blood glucose level of 126 mg/dL or higher. Metabolic syndrome was defined as the presence of at least three of the following components: waist circumference greater than 88 cm, HDL-c level less than 50 mg/dL, TG level of 150 mg/dL or higher, blood pressure greater than or equal to 130/85 mmHg, and glucose level of 100 mg/dL or higher.

#### Quality of life

Quality of life was assessed in the third cycle (2010) using the Women’s Health Questionnaire (WHQ) [12]. The WHQ consists of 36 items divided into nine domains: depressed mood, somatic symptoms, memory/concentration, anxiety/fears, sexual behavior, vasomotor symptoms, sleep problems, menstrual symptoms, and attractiveness. A score of 0 (zero) indicates good health status, a score of 50 % indicates regular health status, a score between 50 and 100 % indicates low health status, and a score of 100 % indicates poor health status.

The 12-Item Short Form Health Survey (SF-12), a shortened validated version of the 36-Item Short Form Health Survey (SF-36), was also used as a tool to assess quality of life in this population. It is a generic questionnaire, and scores also range from 0 to 100 %. The SF-12 is derived from the functional health and well-being domain of the SF-36 (physical functioning, role limitations due to physical health problems, bodily pain, general health, vitality, social functioning, role limitations due to emotional problems, and mental health) [13, 14].

#### Psychiatric disorders

From the second cycle onward, the use of medications for insomnia, anxiety, and depression was investigated. In the three cycles, psychiatric disorders were assessed using the 20-Item Self-Reporting Questionnaire (SRQ-20). The SRQ-20 was developed by the World Health Organization to screen for common mental disorders in primary health care settings, and a version of this instrument has been validated for use in Brazil. The questionnaire consists of questions with yes/no answers about symptoms such as sadness, irritability, headache, pleasure in daily activities, crying frequently, decision-making, appetite, sleep disturbance, lack of concentration, sense of usefulness, and fatigue. It is used to screen for minor psychiatric disorders, and a score of 8 or higher indicates a risk of psychiatric disorders [15–17].

#### Physical activity

From the second cycle onward, physical activity was assessed using structured questionnaires. In the second cycle (2001), physical activity was assessed using a previously tested, standardized questionnaire for each type of physical activity, the Modifiable Activity Questionnaire (MAQ), and metabolic equivalents and total caloric expenditure were calculated [18]. Participants were asked about the type and frequency of the activity performed and time spent standing and sitting. The practice of sports after 18 years of age and in the last year was also evaluated by collecting information on the frequency and duration (in minutes) of each activity*.* Participants who reached an energy expenditure of at least 1000 kcal/week (approximately 3.5 h per week in activities such as walking, climbing stairs, swimming, playing sports, and yard work) were considered physically active, while all others were classified as physically inactive [19, 20].

In the third cycle (2010), the International Physical Activity Questionnaire (IPAQ) was used. This instrument was developed to facilitate the cross-national assessment of physical activity and inactivity. The short-form questionnaire (IPAQ-SF) was used to evaluate self-reported physical activity at four intensity levels (vigorous-intensity activity, moderate-intensity activity, walking, and sitting) over the last seven days [21, 22].

#### Pedometer

In the third cycle (2010), habitual physical activity was assessed using a digital pedometer (BP 148; TechLine, São Paulo, SP, Brazil) [7]. The instrument records the number of steps taken per day for seven days. The mean number of steps was calculated by the ratio between the sum of the daily totals and the number of days the pedometer was used [23]. The device was individually configured according to the participant’s weight (kg) and average step length (distance between the heels in cm). Each participant was given a pedometer and instructed on how to properly use the device. Participants were also provided with a step-recording diary, where they were asked to record the total daily number of steps and the time they put on (wake-up time) and took off (before going to bed) the pedometer. Participants were also instructed to go about their usual activities and to remove the pedometer while showering or sleeping and at the end of each day.

#### Anthropometric measurements

At baseline, weight and height measurements were performed on the same day as blood collection and/or ultrasound examination. Measurements were taken without shoes and heavy clothing. Weight was measured using a mechanical scale (Filizola®, Brazil) and height was measured using a stadiometer coupled to the scale.

In the second and third cycles, anthropometric measurements were performed in duplicate and included body weight, height, waist circumference (measured at the midpoint between the lower rib margin and the iliac crest, perpendicular to the long axis of the body, with the participant standing balanced on both feet, approximately 20 cm apart, with arms hanging freely), hip circumference (widest circumference over the buttocks), and waist-to-hip ratio (waist circumference divided by hip circumference). Body mass index was calculated as weight in kilograms divided by the square of height in meters (kg/m^2^) and categorized as < 25.0, 25.0–29.9, and ≥ 30.0 kg/m^2^ [24]. All measurements were taken without shoes and heavy clothing. Interobserver reliability for anthropometric measurements was verified by repeated measurements during the first round of consultations.

#### Skinfolds

In the second cycle, skinfold thicknesses (mm) at the triceps, suprailiac, and subscapular were measured using skinfold calipers (Scientific CESCORF, RS, Brazil – similar to the Harpenden model) to the nearest 0.2 mm. Values were the mean of three measurements for each skinfold. The percentage of total body fat was calculated by the Faulkner formula: percent total body fat = (triceps + subscapular + suprailiac + abdominal skinfolds × 0.153) + 5.783 [25].

### Assays

At baseline, blood was assayed for 17-beta-estradiol (E2), luteinizing hormone (LH), follicle-stimulating hormone (FSH), sex hormone-binding globulin (SHBG), and thyrotropin-stimulating hormone (TSH). E2 was measured using a solid-phase radioimmunoassay kit (Coat-A-Coat kit; Diagnostic Products Corporation, Los Angeles, CA, USA), with a sensitivity of 20.0 pg/mL. FSH and LH were measured by a solid-phase immunoradiometric assay (MAIAclone; Biodata Diagnostics, Rome, Italy), with sensitivity of 0.25 mIU/mL. SHBG and TSH were measured by a solid phase chemiluminescent immunometric assay using the DPC Immulite kit (Diagnostic Products Corporation), with sensitivity of 0.2 nmol/L and 0.002 IU/mL, respectively.

Total cholesterol, high-density lipoprotein cholesterol (HDL-c), triglyceride (TG), and glucose levels were determined by a colorimetric enzymatic method (Architect C800 System; Abbott Laboratories, Abbott Park, IL, USA). Low-density lipoprotein cholesterol (LDL-c) was determined indirectly using the following formula: LDL-c = total cholesterol − (HDL-c + TG/5) [26].

#### Ultrasound

Transvaginal ultrasound was performed with a Toshiba-Tosbee apparatus (Toshiba Corporation, Tokyo, Japan) using a 5.0 MHz transvaginal probe. The maximum transverse (D1), anteroposterior (D2), and longitudinal (D3) diameters of the ovary were measured with electronic calipers. Ovarian volume was calculated using the following formula: volume = D1 × D2 × D3 × 0.523. Ovarian cyst was defined as a lesion with its largest diameter measuring at least 25 mm [27]. The examinations were performed in different phases of the menstrual cycle. Cycle phases were classified as follicular (days 1–10), periovulatory (days 11–17), and luteal (day 18 and later). The association of ovarian volume with menstrual cycle phase was analyzed to minimize any potential bias resulting from the effect of cycle phase on ovarian volume [27–29].

At baseline, all ultrasound examinations were performed by the same examiner. In the second cycle (2001), examinations were also performed by a single examiner and the interobserver correlation coefficient was calculated by comparing results of each case between the first- and second-cycle examiners. Reproducibility of the ovarian volume measurement was evaluated using the intraclass correlation coefficient to calculate the level of agreement with a second observer. Ovarian volume measurement achieved excellent reproducibility, with an intraclass correlation coefficient of 0.957 (95 % CI 0.883–0.94) for the right ovary and 0.982 (95 % CI 0.940–0.994) for the left ovary.

#### Densitometry

In the third cycle (2010), bone mineral density was assessed in the lumbar spine (L1-L4), femoral neck, and proximal total femur by dual-energy X-ray absorptiometry (Lunar Prodigy Advance DXA System; GE Medical Systems, Milwaukee, WI, USA) in all participants. The results were expressed in g/cm^2^ and estimated z- and t-scores. All measurements were performed in the Division of Radiology at Hospital São Vicente de Paulo.

#### Computed tomography (CT)

The coronary artery calcium score was assessed by chest CT using a 128-channel multidetector CT scanner (Definition; Siemens Medical Systems, Erlangen, Germany) at Hospital São Vicente de Paulo*.* The same radiologist read all CT scans at a Siemens Workstation (Leonardo 4.1). The average Agatston score was used in all analyses [30]. Subcutaneous and visceral fat areas were measured on a cross-sectional scan obtained at the umbilicus (L3-L4) as previously described [31].

#### Mortality data

In the third cycle (2010), medical records were reviewed to collect information on age at death, date and cause of death. The causes of death were coded according to the International Classification of Diseases, 10th revision [32].

## Discussion

The study findings provide relevant information on health issues in midlife women and allow us to evaluate the association between menopausal status, aging in women and the risk of metabolic comorbidities and cardiovascular diseases in a representative sample of women from southern Brazil. Key publications to date are summarized in Table 2.

**Table 2:** Main results of the PFS

Author, year	Study design	N participants	Objective	Conclusion
Oppermann et al. 2003 [33]	Cross-sectional	98	To evaluate the relationship between ovarian volume and age, hormone levels, obesity, and menstrual cycle phase in pre- and perimenopausal women.	Ovarian volume was smaller in pre- and perimenopausal women aged 40 years or older compared with younger women.
Bastos et al. 2006 [5]	Cross-sectional	273	To investigate the association of smoking, parity, BMI, oral contraceptive use, and hormone therapy with ovarian volume in pre-, transition, and postmenopausal women.	Obesity was positively related to ovarian volume, menopausal status, and age. Use of contraception was associated with reduced ovarian volume.
Donato et al. 2006 [6]	Cross-sectional	358	To investigate the association between menopausal status and central adiposity measured by two different cutoffs of waist circumference and waist-to-hip ratio.	Postmenopausal women were at greater risk of having central adiposity (waist circumference and waist-to-hip ratio) than premenopausal women.
Oppermann et al. 2012 [15]	Cross-sectional	324	To identify the prevalence of physical, psychological, and menopause-related symptoms and their association with minor psychiatric disorders in pre-, peri-, and postmenopausal women.	Low level of education, memory loss, irritability, and menopausal transition were risk factors for positive findings in screening for minor psychiatric disorders.
Colpani et al. 2012 [7]	Cross-sectional	292	To assess pedometer-determined habitual physical activity in a Brazilian cohort of pre-, peri-, and postmenopausal women and its effect on anthropometric measurements and cardiovascular risk factors.	Walking 6,000 or more steps daily was associated with a decreased risk of CVD and DM in middle-aged women, regardless of menopausal status.
Colpani et al. 2014 [34]	Cross-sectional	292	To compare two methods of assessing physical activity in pre-, peri-, and postmenopausal women.	The agreement (k = 0110; *p* = 0.007) and correlation (rho = 0.136, *p* = 0.02) between the IPAQ-SF and the pedometer were weak.
Colpani et al. 2014 [35]	Longitudinal	358n	To assess mortality rate, causes of death, and associated risk factors in climacteric women.	CVD was an important cause of death in this cohort. DM and/or central adiposity were associated with all-cause mortality.

*BMI* body mass index, *CVD* cardiovascular disease, *DM* diabetes mellitus, *IPAQ-SF* International Physical Activity Questionnaire-Short Form

## Abbreviations

ABIPEME: Brazilian Association of Market Research Institutes
BMD: Bone mineral density
CAC: Coronary calcium score
CT: Computed tomographic scanning
CV: Cardiovascular
DXA: Dual-energy X-ray absorptiometry
E2: 17-beta-estradiol
FSH: Follicle-stimulating hormone
HDL-c: High-density lipoprotein cholesterol
HT: Hormone therapy
IBGE: Brazilian Institute of Geography and Statistics
IPAQ: International Physical Activity Questionnaire
LDL-c: Low-density lipoprotein cholesterol
LH: Luteinizing hormone
MAQ: Modifiable Activity Questionnaire
NCDs n: Non-communicable diseases
NIS/RS-SES: Center for Health Information
PFS: Passo Fundo Cohort Study
SF-12: 12 Item Health Survey
SF-36: Short Form Health Survey -36
SHBG: Sex hormones binding globulin
SRQ-20: Self-Reporting Questionnaire
TG: Triglyceride
TSH: Thyrotrophin stimulating hormone
WC: Waist circumference
WHQ: Women’s Health Questionnaire
WHR: Waist-to-hip ratio
